# Analysis of a comprehensive dataset of diversity generating retroelements generated by the program DiGReF

**DOI:** 10.1186/1471-2164-13-430

**Published:** 2012-08-28

**Authors:** Thomas Schillinger, Mohamed Lisfi, Jingyun Chi, John Cullum, Nora Zingler

**Affiliations:** 1 Department of Molecular Genetics, University of Kaiserslautern, Kaiserslautern, Germany; 2 Department of Genetics, University of Kaiserslautern, Kaiserslautern, Germany; 3 Department of Biology - Group of Molecular Genetics, University of Kaiserslautern, Paul-Ehrlich-Straße Building 24, Room 117, D-67663, Kaiserslautern, Germany

**Keywords:** DGR, Diversity-generating retroelement, Targeted mutagenesis, Prokaryote evolution, Horizontal gene transfer, Reverse transcriptase, DiGReF

## Abstract

**Background:**

Diversity Generating Retroelements (DGRs) are genetic cassettes that can introduce tremendous diversity into a short, defined region of the genome. They achieve hypermutation through replacement of the variable region with a strongly mutated cDNA copy generated by the element-encoded reverse transcriptase. In contrast to “selfish” retroelements such as group II introns and retrotransposons, DGRs impart an advantage to their host by increasing its adaptive potential. DGRs were discovered in a bacteriophage, but since then additional examples have been identified in some bacterial genomes.

**Results:**

Here we present the program DiGReF that allowed us to comprehensively screen available databases for DGRs. We identified 155 DGRs which are found in all major classes of bacteria, though exhibiting sporadic distribution across species. Phylogenetic analysis and sequence comparison showed that DGRs move between genomes by associating with various mobile elements such as phages, transposons and plasmids. The DGR cassettes exhibit high flexibility in the arrangement of their components and easily acquire additional paralogous target genes. Surprisingly, the genomic data alone provide new insights into the molecular mechanism of DGRs. Most notably, our data suggest that the template RNA is transcribed separately from the rest of the element.

**Conclusions:**

DiGReF is a valuable tool to detect DGRs in genome data. Its output allows comprehensive analysis of various aspects of DGR biology, thus deepening our understanding of the role DGRs play in prokaryotic genome plasticity, from the global down to the molecular level.

## Background

Living organisms utilize many mechanisms to ensure fidelity of replication and to reduce the mutation rate. However, in some circumstances, an increased mutation rate can be beneficial. In particular, pathogenic organisms are often subjected to selection for diversity to overcome host defenses and/or increase host range. For example, mutator mutants lose the mismatch repair system [1], which affects the entire genome. Alternatively, changes in the copy number of simple repeats at bacterial contingency loci can generate high frequencies of mutations in particular genes [2], but result in a limited range of potential mutations. Diversity generating retroelements (DGRs) can generate a much greater range of localized diversity. The first DGR was discovered in a *Bordetella* phage, where it affects tail fibers and, thus, host range [3]. Since then, DGRs have been discovered in a variety of phage and bacterial systems [4-6].

**Figure 1:**
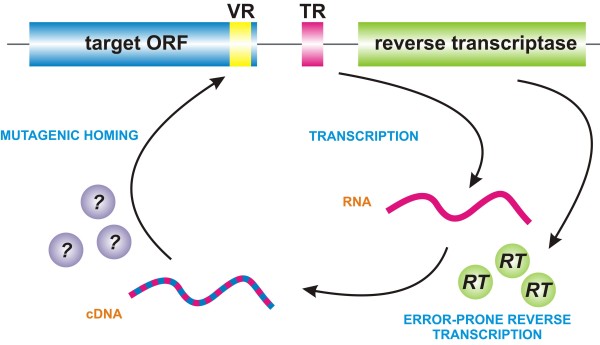
**Mode of action of diversity-generating retroelements (DGRs).** DGRs always comprise an ORF encoding a reverse transcriptase (RT), a template repeat (TR) and at least one target ORF harboring the variable region (VR), which corresponds to the TR. First, an RNA transcript is made from the TR, which is then reversely transcribed by the RT in an error-prone fashion. In a process termed mutagenic homing, the mutagenized cDNA replaces the parental VR in the target ORF, thereby altering the host gene.

DGRs include a gene encoding a reverse transcriptase (RT) as well as a template repeat (TR) and a variable repeat (VR) (Figure 1). The VR is expected to lie within a protein coding region, so that mutagenesis results in an altered protein sequence (the tail fiber protein in the case of the *Bordetella* phage). In the known DGRs, the TR/VR repeats are about 120 bp long. The sequence of the TR can be copied in an error-prone fashion to the VR resulting in the generation of diversity. A hallmark of DGRs is the exclusive mutation of sites that correspond to adenine residues in the respective template repeat. There is convincing experimental evidence that mutagenesis occurs through reverse transcription of a cDNA from a transcript containing the TR [3,7]. This process is thought to resemble the target-primed reverse transcription mechanism also employed by group II introns and non-LTR retrotransposons. However, instead of the “copy and paste” mechanism of these classical retroelements, DGRs use a “copy and replace” strategy. Since the system does not self-inactivate, it is able to generate continuous localized mutagenesis. Although recently a specific structure in the DNA close to the VR has been identified as a crucial targeting determinant [8], the exact steps of the exchange of the genomic DNA for the newly generated cDNA are still unclear. The reason for the A-specific nature of the base changes is also unknown. The RTs associated with DGRs seem to belong to a unique clade most closely related to group II intron RTs [4]. The best investigated DGR includes a second small protein encoded by the *atd* gene, which is important for mutagenesis. However, the function of the protein and whether such a protein is required for all DGRs is unclear. DGRs occur in a taxonomically diverse range of bacteria and phages [5,6]. Little is known of their evolution, including whether they have evolved via horizontal gene transfer (HGT) and how they acquire new target proteins.

The DGR characteristics described above have been mainly derived from investigation of a single element, the *Bordetella* phage DGR, and supported by sequence comparison with a small number of related elements. However, a systematic and comprehensive assessment of the prevalence, distribution, and structure of these retroelements has been lacking. In this paper, we present a Perl program that identified 155 potential DGRs in public DNA sequence databases, the largest set described so far. Having subjected this dataset to careful quality control, we used it to examine several aspects of DGR mechanism and evolution. We found that DGR cassettes have a rather homogenous length of 2–5 kb, but are highly tolerant to permutations of their components and expansion with up to three additional VRs. TR and VR can even be on a different DNA strand to the corresponding RT gene. Thus, unlike in group II introns and retrotransposons, the RT mRNA and the template RNA are not necessarily the same molecule. DGR RTs, though highly divergent, form a phylogenetic clade that is characterized by a (I/V/L)GxxxSQ motif in RT domain 4. This motif seems largely necessary and sufficient to predict DGR association and may explain the observed restriction of mutagenesis to adenine bases. DGRs can be found in all major classes of bacteria, but exhibit sporadic phylogenetic distribution. Several lines of evidence point to horizontal gene transfer as the main propagation mechanism of DGRs. However, DGRs do not use a single vector for their dispersal, but “hitchhike” with various mobile elements, e.g. phages, transposons and plasmids.

## Results and discussion

### DiGReF reliably identifies potential DGRs

The sequences in the NCBI nr protein database were subjected to a psi-blast search to identify sequences that potentially encode RT enzymes. There were 2651 hits. DNA Sequences 5000 bp up- and downstream of each RT were extracted and subjected to analysis by the DGR-finder program DiGReF (Additional file 1). This algorithm uses a sliding window (default size 50 nucleotides), which is used to search the complete extracted sequence for repeats of its sequence. To account for the characteristics of DGRs, all non-A bases in the window have to match exactly, but the adenines in the window do not have to match. When such a hit is found, it is extended to yield the maximum length sequence in which all non-A bases match. The program designates the sequence derived from the search window as the template repeat (TR), and the mutated repeat as the variable region (VR).

To eliminate artifact hits such as low complexity repeats or sequences that are a result of recent gene duplication events, only repeats with at least 10 adenines in the TR and at least 7 A → B substitutions in the VR (B = G, C or T) were considered. TR sequences with less than 10 potential mutation sites would only be able to provide a diversity of < 2.6 x 10^5^ possible VR sequences. Due to the logarithmic correlation between repertoire size and the probability of finding a protein of the desired properties in a repertoire [9,10], repeats with low diversification potential are more likely artifacts (e.g. group II intron RTs associated with random repeat-like sequences) than efficient DGRs. Manual inspection of samples confirmed this assumption.

With these criteria, 155 of the 2651 RT hits could be identified as containing DGR-like repeat structures, 126 of which had not been previously described (Additional file 2A). VR/TR hits were overwhelmingly found associated with RTs that have a high homology to known DGR RTs. To explore more distantly related RT sequences, we performed further iterations of psi-blast with newly found DGR RTs that were more dissimilar to the “standard” DGR RTs. However, we did not detect additional DGRs, and we are thus confident that we have reached saturation and that our dataset is comprehensive. This strategy also served as a test to assess the possibility that VR/TR-like repeats are widely abundant in genomic sequences and thus also often found in the vicinity of RT genes by chance. Using a cut-off of seven A substitutions, we did not find such fortuitous repeats. However, lowering the cut-off to five A exchanges resulted in 41 additional hits which upon manual inspection seemed mostly false positives (Additional file 2B). Still, six of these hits match the known characteristics of DGRs, but are lost in the higher cut-off setting. Depending on the objective of the user, it is thus possible to emphasize detection sensitivity or stringency of the DiGReF program by adjusting the cut-off values. In this paper, we wanted to avoid as many false positive hits as possible and thus carried out further analyses with a cut-off of seven A substitutions. The few false positives and false negatives that remained are discussed later in the text.

In addition to the coordinates and sequences of the VR/TR pairs, the program also delivers an alignment of the repeats, statistical data on the adenine exchanges, and an annotation file that can be opened in a sequence viewer such as Artemis [11] to visualize the DGR structure (Additional file 3). Due to its modular nature, the software can be easily adapted and expanded to address other questions that might arise while DGRs are being studied in more detail.

### DGRs are ubiquitous among prokaryotes

**Figure 2:**
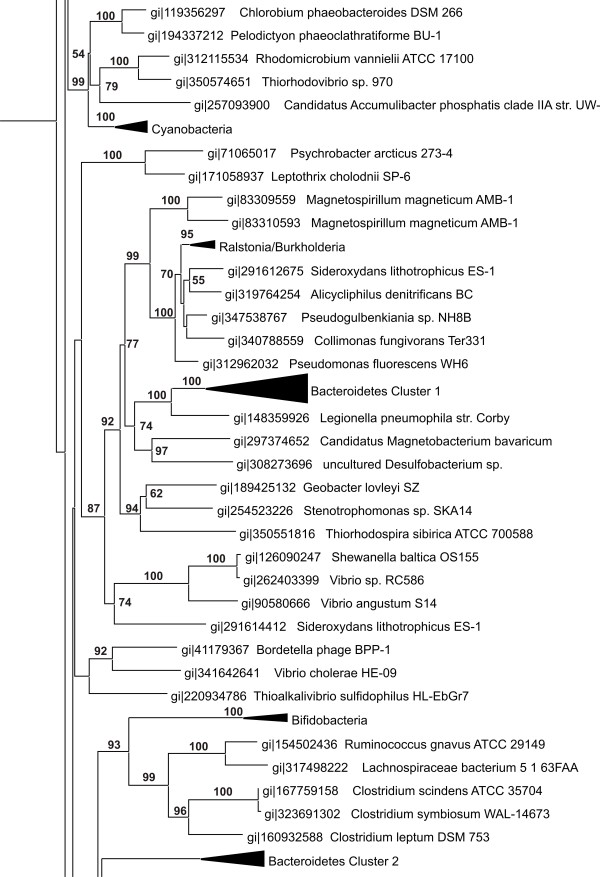
**Phylogenetic Tree of DGR RTs (representative selection).** A phylogenetic tree was compiled using a Neighbour-Joining algorithm, bootstrapping was done with 1000 replications using PHYLIP. Groups of DGR RTs that form highly uniform clades with high bootstrapping values are shown collapsed. The complete tree is supplied online as Additional file 2.

It has been reported previously that diversity-generating retroelements are found in all major prokaryotic classes [4]. Our data corroborate and significantly expand this finding. Even in the systematic search covering the complete non-redundant NCBI database, DGRs were never associated with eukaryotes or archaea. We identified DGRs in some phages and all but the smallest prokaryotic NCBI taxonomic classes, with the majority associated with the Bacteroidetes and Firmicutes classes (Table 1). Due to the low number of sequenced organisms in the minor classes (they only make up four percent of the available prokaryotic sequences), it is possible that further sequencing efforts may reveal DGRs here as well. Also, a sampling bias may cause an apparent overrepresentation in a certain genus, such as many similar *Bacteroides* entries that stem from different patient isolates. Therefore, a reliable quantitative assessment of DGR distribution across prokaryotic phyla is not possible at the moment. However, several qualitative conclusions can be drawn from these data: DGRs are widespread or even ubiquitous among prokaryotes. Still, their prevalence is rather low (155 DGRs in >6000 sequenced organisms). Moreover, their distribution is clearly paraphyletic, i.e. they are not ubiquitous in any bacterial group. This may be due to repeated independent losses of DGRs in related species, or due to horizontal gene transfer (HGT) by mobile genetic elements. To distinguish between these two possibilities, phylogenetic trees were constructed using the amino acid sequences of RTs from DGRs (Figure 2 and Additional file 4). In most cases, the phylogenetic trees of RTs were compatible with those of their host organisms (deduced from NCBI taxonomy and a 16S tree, Additional file 5). However, in some cases, RT clades contained distantly related host organisms, strongly arguing in favor of HGT. Below, the genomic context of several such cases is analyzed in more detail.

**Table 1:** Phylogenetic distribution of DGRs

**Classification**	**Sequenced genomes on NCBI [%]**	**Hits in our dataset [%]**
Actinobacteria	7,8	5,2
Bacteroidetes/Chlorobi group	3,9	27,7
Cyanobacteria	2,3	5,8
Deinococcus-Thermus	0,6	0,6
Firmicutes	23,6	31,0
Nitrospirae	0,1	0,6
Alphaproteobacteria	9,0	2,6
Betaproteobacteria	6,0	7,7
Gammaproteobacteria	21,4	12,3
Delta/Epsilonproteobacteria	3,1	2,6
Spirochaetes	5,6	0,6
unclassified Bacteria	0,2	0,6
Chlamydiae/Verrumicrobia group	1,0	0,6
Phages	11,7	1,9
Other Bacteria	3,8	

### DGRs use different mechanisms to transfer between species

**Figure 3:**
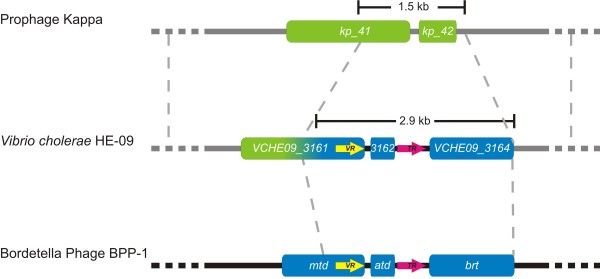
**Core DGR element found in *Vibrio cholerae *HE-09.** A 1.5 kb fragment of the *Vibrio* phage kappa was replaced by a 2.9 kb fragment in *V. cholerae* HE-09, including an RT ORF, a template repeat (TR), an *atd* ORF and an *mtd* ORF including a variable region (VR), The inserted fragment bears high homology to corresponding elements in the *Bordetella* phage BPP-1. Sequences upstream and downstream of the 2.9 kb element correspond to homologous sequences in *Vibrio* phage kappa.

Since DGRs were first identified in a bacteriophage [3], phages are obvious candidates for HGT vectors of DGRs. However, we found surprisingly few additional examples of DGRs in phage genomes, despite the abundance of sequenced phages (over 700 bacteriophages) in the database. Attempts to test association of DGRs with prophages using the program Prophage Finder [12] were not successful. However, we found a striking example of phage-mediated DGR transfer in *Vibrio cholerae* HE-09. The reverse transcriptase of *V. cholerae* HE-09 shares a clade with the DGR RT from *Bordetella* phage BPP-1. The neighbouring genes encode proteins similar in sequence to Atd and Mtd (Figure 3). When the 72 kb contig containing the *V. cholerae* HE-09 RT was used for a blastn search, it showed extensive homology to the kappa prophage of *V. cholerae* B33. About 1.5 kb of the kappa sequence is replaced by 2.9 kb containing the DGR, resulting in a fusion between the putative kappa tail fiber protein gene and the *mtd* gene (Figure 3). This structure was most likely formed by recombination of BIP-1 or a related phage with a kappa phage, which then in turn integrated into the *V. cholerae* HE-09 genome. The 2.9 kb region comprising RT, *atd*, TR, VR, and part of the *mtd* thus constitutes the smallest functional DGR unit with evidence of direct physical transfer between species. The target ORF seems to be a preferred target of recombination since we found three other instances of fused target genes in HGT events (data not shown).

A different transfer strategy was used in the case of the DGRs from *Vibrio* sp. RC586 (GI 262403399) and *Shewanella baltica* OS155 (GI 126090247). These DGR cassettes show an overall sequence identity of 93%. In *Shewanella*, the element is located on plasmid pSbal02, which itself can potentially be exchanged between organisms. Moreover, the DGR is close to a transposase/integrase gene, which may be responsible for mobilization of the whole element. In *Vibrio* sp. RC586, the DGR is located in the vicinity of Tn7-type sequences, exactly at the position where Tn7 usually carries antibiotic resistance genes. DGRs therefore may be mobilized by transposons and might even co-opt the same integron system that appears to exchange resistance markers in complex transposons [13].

We could also observe transfer events encompassing DGRs in *Bacteroides* species. These human gut bacteria are known to have a very plastic genome and a plethora of autonomous and non-autonomous mobile elements that are transferred mostly by conjugation [14]. They also carry numerous DGRs that cluster in two separate clades according to the RT phylogeny (Figure 2). For example, *Bacteroides* sp. 1_1_14 harbors two DGRs, one each from the two Bacteroidetes clusters in the phylogenetic tree (Additional file 4). One of these DGRs is located on a 42.6 kb fragment that is 99% identical to a *B. ovatus* 3_8_47FAA sequence, but the flanking sequences display 95–99% identity with the genome from *B. thetaiotaomicron* VPI-5482. The nature of the 42.6 kb fragment is not clear. A direct blastn query does not result in significant hits, but one of the encoded proteins is homologous to transposition proteins, thus suggesting a conjugative transposon or a transposable phage as shuttle for the DGR element.

These examples suggest that DGRs do not have a dominant mode of interspecies transmission. They can use bacteriophages, plasmids and transposons for dispersal. The selective advantage they provide to the host should help them to stay maintained in those gene transfer vectors.

### DGR reverse transcriptases form a distinct and well-defined clade characterized by an SQ dipeptide

**Figure 4:**
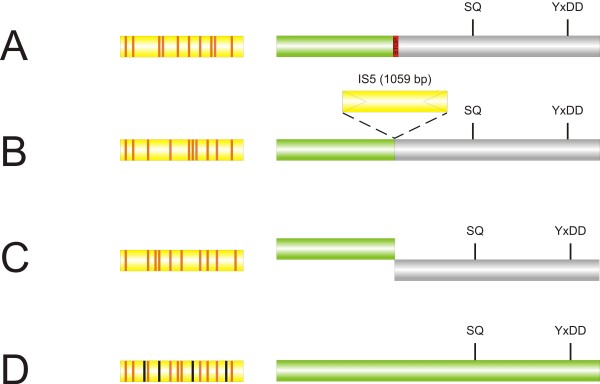
**Inactivated or unusual DGRs.** We encountered several examples of DGRs with inactivated RTs, but intact VR/TR repeats. This includes (A) nonsense mutations as in *Ruminococcus gnavus* ATCC 29149, where a premature stop codon truncates the RT ORF, (B) disruption of the RT ORF by insertion elements as in *Acaryochloris marina* MBIC11017, or (C) frameshift mutations as in *Bacteroides sp.* 9_1_42FAA. (D) Some DGRs contain full-length RT ORFs, but several B-to-N mutations in their VR. VRs of the respective elements are shown on the left and depicted in yellow (A-to-B mutations: orange vertical bars; B-to-N mutations: black vertical bars). The corresponding RTs are schematically shown on the right (not to scale). The 5’ part of the RT is shown as green box, inactivated parts in gray.

Using the set of potential DGRs identified by DiGReF, we next examined the corresponding RTs in further detail. Their size ranged from 260 to 527 aa. We found a few shorter sequences, but upon manual inspection, these entries proved to be RT genes that had been truncated by a mutation event (a transposon insertion, a nonsense mutation or a frameshift mutation, Figure 4A-C). It is possible that the apparent point mutations arise because of sequencing errors and that the DGRs are actually intact. However, it seems unlikely that all apparent inactivation events (including transposon insertions) can be explained in this way. The inactivation of the RTs must have happened very recently, since the corresponding VR/TR pairs harbored only A-mutations, not additional random mutations as would be expected to accumulate in an inactivated DGR.

The average length of 378 aa was in line with the 377 aa reported as average DGR RT length by [5]. An alignment of all identified DGR RTs showed the clear organization into seven conserved domains (Additional file 6) that had been described before for a smaller subset [5]. Following region 7, we noticed a patch of 20 amino acids that is highly positively charged (often 50–60% R and K residues, Additional file 7) Although there is no distinct pattern discernible in the arrangement of charged residues, this positively charged region appears to be unique to DGR RTs. This C-terminal region likely is involved in nucleic acid binding, for example in template recognition.

**Figure 5:**
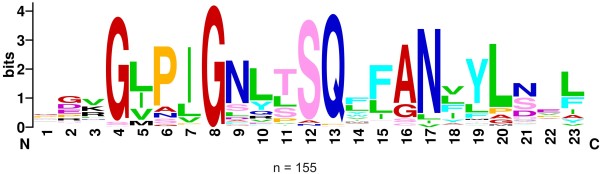
**Sequence logo of motif 4 of DGR RTs.** A total of 155 substrings comprising motif 4 of the DGR RTs were taken from our result set and a sequence logo was created using WebLogo [40,41]. Numbers 1 to 23 on the x-axis indicate the relative position in the substring. The height of the symbols denotes the relative frequency of each amino acid at the respective position, while the overall height of the stack represents the degree of conservation measured in bits.

One of the most prominent features of DGR RTs is the characteristic (I/V/L)GxxxSQ motif in region 4, subsequently referred to as SQ motif in this text (the alignment shown in Additional file 6 is summarized as sequence logo in Figure 5). It is highly conserved and differs from the QGxxxSP motif found in most retroviral and non-LTR-retrotransposon RTs and group II intron maturases (Additional file 6) [5,15]. The SQ motif had been associated with DGRs before [5,6,8], but due to the small sample size, it was not clear whether it was common to all DGR RTs. Our systematic screen revealed that the SQ motif is conserved in 90 % of the identified DGRs (Additional file 2A). In fact, in the (L/I/V)GxxxSQ motif, the underlined portion is almost invariant, while the last two residues vary in only 15 of 155 elements (SP, SH, NQ, PA, AQ, VQ) (Figure 5). Despite these variations, the associated DGRs display adenine exchanges in their respective VRs and contain a target ORF, suggesting that they are functional. Therefore, the SQ dipeptide may not be absolutely necessary for DGR activity; however, as in the case of the truncated RTs discussed above, the apparent mutations might be sequencing errors or very recent inactivations.

### Apparent exceptions from the DGR RT clade are often artifacts

Twenty-eight sequences of the input sequences that include the SQ motif did not produce hits with DiGReF (Additional file 2C). Apart from six RTs that are clearly truncated and thus not functional, they are most likely false negatives. They are apparently intact, featuring SQ and YxDD motifs, but are not associated with obvious TR/VR repeats. Many are located on short contigs of less than 5 kb, or very close to the end of a contig. Thus, the complete DGR sequence is not included in the program input, making it impossible to identify VR/TR repeats. In at least two of the remaining sequences (Figure 4D), we could manually identify repeats that include several non-A mutations and therefore cannot be identified by DiGReF. They may be “sloppy” elements that are still active with a reduced A-specificity, but the high number of non-A mutations could also suggest that these hits represent DGR elements that are no longer functional. Selective pressure for high DGR activity might decrease once the target protein is well adapted to its function, thus fixing it in the genome with a normal mutation rate. Decreasing the window size for repeat scanning would allow detection of such “sloppy” repeats, but would also lead to more false positive hits.

### The SQ motif may be responsible for RT mediated mutagenesis

Our comprehensive search showed that DGRs are only found within the subset of RTs that cluster with already known DGR RTs. Considering that DGR cassettes are otherwise highly diverse in structural organization, accessory proteins and VR-ORFs (see below), this monophyletic origin means that the RT function in DGRs cannot easily be replaced by another bacterial (group II intron) or viral RT, arguing for the involvement of the RT in diversification. If host factors were responsible for editing the RNA or cDNA, the high plasticity of bacterial genomes would make RT swaps quite likely. The exclusive association with the SQ-clade of RTs prompted us to analyze the most highly conserved regions 4 and 5 for possible structure/function relationships.

The catalytically essential aspartate residues of reverse transcriptases are located in domain 5. In DGR RTs, they are part of a YxDD motif. While the two aspartates are 100% conserved, the tyrosine is replaced by phenylalanine in 14% of the cases. The second position is not that highly conserved. 53% of the DGR RTs have an M at this position, 33% a V, and the remaining entries feature the small non-polar amino acids A or C (Figure 5). In HIV RT, the corresponding M184V mutation has a strong influence on the fidelity of the reverse transcriptase [16,17]. Therefore we analyzed whether DGRs that carried a V instead of an M in domain 5 displayed an altered mutation pattern, but we did not find significant differences either in the overall mutation rate (mutated adenines per total number of adenines in the TR) or in the distribution of mutated nucleotides (data not shown).

The almost exclusive appearance of the SQ motif in region 4 of DGR RTs suggests a mechanistic connection between these amino acid residues and the function of DGRs. Since the unique feature of DGRs is adenine-specific mutagenesis, we hypothesize that the SQ motif plays a vital role in defining RT fidelity. The crystal structure of HIV RT in complex with a DNA template:primer and a dNTP has suggested that domain 4 (which comprises the QGxxxSP motif) participates in binding and selection of the incoming nucleotide as well as template coordination near the active site [18]. In HIV RT, mutation of Q151 changes the discrimination between rNTPs, dNTPs and ddNTPs, the activity on DNA and RNA templates, and the fidelity of the polymerase [19-21]. P157, which corresponds to the Q in region 4 of DGR RTs, is considered part of the template grip; mutations in this residue also affect nucleotide incorporation patterns [22,23]. Thus mutations in motif 4, coordinating both template and incoming dNTPs, seem ideally poised to modify RT fidelity. As DGR RTs only have relaxed fidelity at As, changes in the binding pocket may specifically modify the interaction with adenine residues. For example, flipping out the template bases in the active site, a process that has been observed in many polymerases [24,25] may be disturbed in DGR RTs. The geometry of template adenine coordination may be altered to make the enzyme more welcoming for non-complementary incoming nucleotides. Thus, region 4 may be responsible for misincorporations in the resulting cDNA which could lead to the observed A → B mutations on the VR coding strand.

### Nucleotide substitutions are essentially random

**Figure 6:**
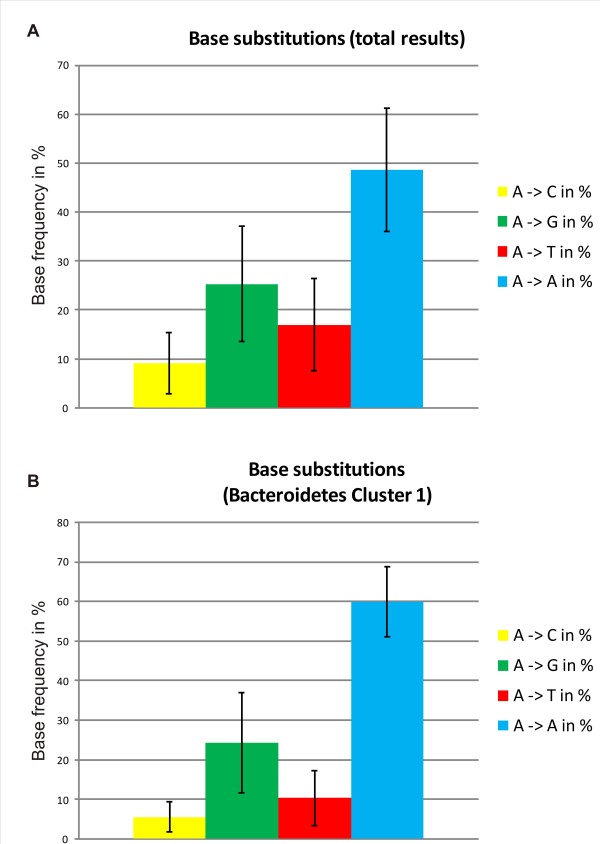
**Statistical analysis of adenine substitutions.** Frequencies of the four bases were determined for each adenine-corresponding position in the VRs of our data set. Bars represent the mean values of these frequencies over the total results set (**A**) or the *Bacteroides* cluster 1 (**B**). Error bars are +/− standard deviation. The frequencies of the three nucleotides C, T and G in (**A**) were significantly different from each other (p < 0.001, χ^2^ test).

To further analyze the pattern of misincorporations, we determined the frequency of each of the four bases at the variable positions in the VRs of all identified 155 elements (Figure 6A). Usually, the majority of the A-residues present in the TR are unchanged in the VR, but this fraction is highly variable (13–79%). It is inherently impossible to distinguish A residues that were generated by exact base pairing from A residues that were included through random incorporation of any nucleotide at that position. At the mutated positions, transitions to guanine were most common, followed by the transversions, with C substitutions being rarest (Figure 6A). Individual VRs had widely varying ratios of substituted nucleotides.

It seems likely that host factors or different selectivity of individual RTs might influence the choice of substituted nucleotides. If there were no such selectivity, the average number of changes to each nucleotide in a VR would be proportional to the number of A-residues in the TR. The observed results were compared with these predictions using χ^2^ tests. Significant differences were found for G-residues (p = 0.00008) and T-residues (p = 0.0057), but not for C-residues (p = 0.13). This suggested some influence of host factors or RT specificity on exchange preferences. To investigate this further, we selected a group of 15 DGRs that carry closely related RTs and are found in one genus, the *Bacteroides* (see Figure 2). We analyzed whether these DGRs exhibit a stronger or more homogenous substitution bias. However, we found comparable distribution patterns with equally high variability as in the complete dataset (Figure 6B). Even individual VRs within a DGR containing multiple VRs (see below) can have drastically different exchange patterns (data not shown). Thus, our data argue against a strong structural or enzymatic bias for nucleotide selection opposite As in the RNA template. However, the genomic sequences are a snapshot of DGR mutagenesis biased by selective pressure. It is possible that in addition to functional selection bias, differences in %G + C-content and thus, in codon usage, could lead to different biases in different classes of organism. Such questions might be partly addressed by examining the effects of the mutations on the codon affected. However, to truly understand the underlying mechanism, individual DGRs will have to be studied experimentally in more detail.

### Repeat length is limited to ~ 150 bp

By providing automated retrieval of sequence information and assignment of VRs and TRs, DiGReF allows for comprehensive analyses of structural features of DGRs. For example, comparison of VR lengths showed that most VRs lie in the range of 100 ± 50 bp. Upon manual inspection, the shorter TR/VR pairs are often flanked by a non-A mismatch and can usually be extended further, but they never exceed 180 bp (data not shown). The relatively short repeat length is in line with recent experimental evidence which showed that although DGRs tolerate some extra sequence in their TR and can transfer it to the VR, longer additional DNA sequences are quickly purged [26]. The observed restriction of the repeat length could be due to low processivity of the RT or a specific recombination mechanism that favors exchanges of shorter DNA stretches. However, it is also possible that the process is not limited mechanistically, but functionally: if the resulting protein loses activity when larger patches of its sequence are hypermutated, there would be a strong selective pressure to keep the VRs short.

### DGR structure is highly variable

**Figure 7:**
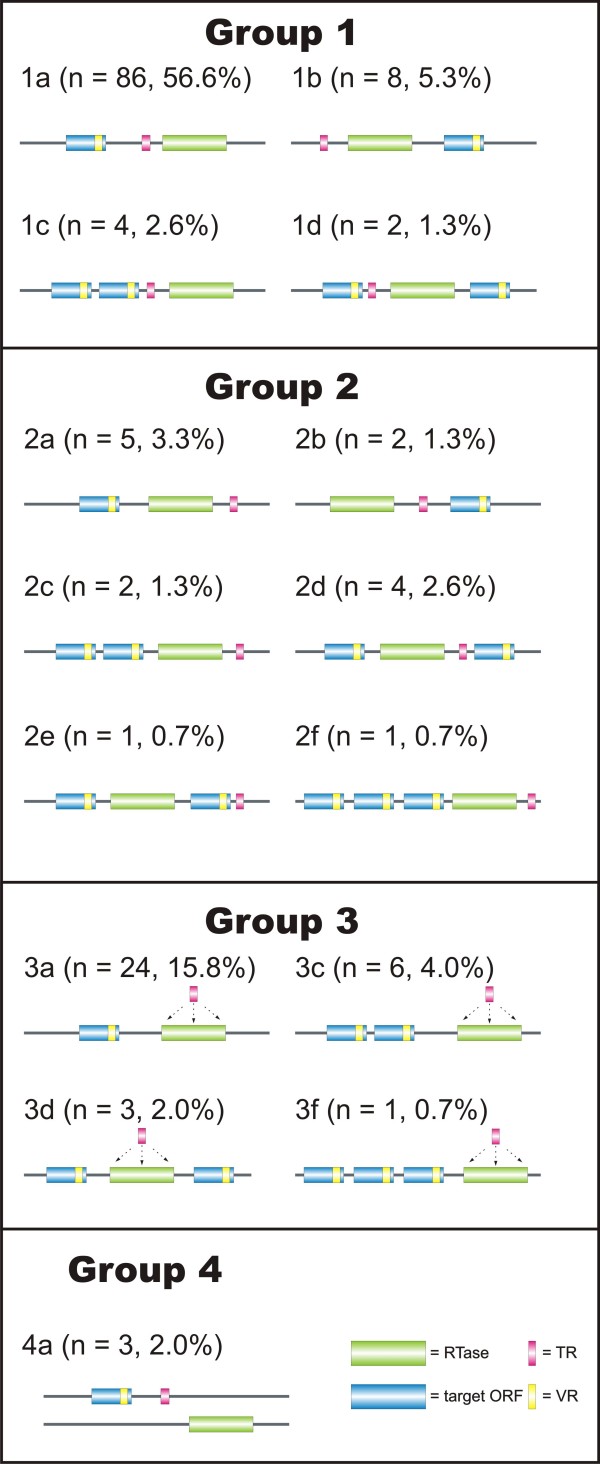
**Structural diversity of DGRs.** Arrangements of DGR elements was determined and DGRs were classified into four main groups and respective subgroups. The number and fraction of hits per subgroup is given in parentheses. RT ORFs are shown in green and target ORFs in blue, TRs are depicted in pink and VRs in yellow. Some subgroups (1e, 1f, 3b, 3e, etc.) were not observed in our data set and are thus missing in this figure.

Previous investigations of DGRs had noted considerable variation in the relative order of cassette components [4,6]. Therefore, we implemented a module in DiGReF that converts the results into a graphical output, and analyzed all DGRs in our final dataset for their cassette architecture. The arrangement of the cassette components shows no strict requirement for a certain order. The majority of DGRs (56.6%) follows the pattern described for the prototypical *Bordetella* phage DGR (1VR-TR-RT) [3]. We classified the structures based on the relative position of TR and RT (Figure 7). Group 1 carries the TR upstream of the RT, in group 2 the TR is located downstream of the RT, and in group 3, the TR overlaps with the 5’- or the 3’-end of the annotated RT ORF. A previously unknown cassette structure, where the TR and RT are located on different strands (see below), was classified as group 4. Within those groups, we observed a large variety of arrangements. Using the number and position of the target ORFs as secondary classification criteria, we established subgroups indicated by small roman letters (a, b, c,…). This system allows for accommodation of additional structure types that may be identified through future sequencing efforts.

DGR structure does not seem to be tightly correlated with RT phylogeny. For example, structural group 2 consists mostly of cyanobacterial sequences with similar RTs, but also includes other elements, e.g. from *Haliscomenobacter*, which have highly divergent RTs (Additional file 4). Also, we observed that different structural DGR types can persist in parallel within one class of bacteria and sometime within one organism, e.g. in *Candidatus accumulibacter*. The absence of a strict order in the cassette components implies that the spatial arrangement is irrelevant to the DGR mechanism. The RT- and TR-RNAs are most likely separately transcribed, and homing is an independent process.

### A significant fraction of DGRs includes multiple VRs

While most DGRs had the described standard structure comprising one VR, a TR and the RT [6], we found 19 instances of DGRs with two VRs, two examples with three VRs, and, after extending the analyzed region, even three examples with four VRs (Table 2). Thus, roughly a seventh of the DGRs from our dataset were identified to comprise multiple VRs. To make sure that the additional VRs in multiple repeat DGRs were not merely random hits created by chance, we confirmed that most VR sequences were indeed part of an ORF. In only two instances, a clear assignment of a VR to an ORF was not possible. In *Pelodictyon phaeoclathratiforme* BU-1, one of the four VRs is not part of an identifiable coding region, whereas in *Marichromatium purpuratum* 984, all three VRs belong to a single 3366 bp ORF that consists of three tandemly arranged FGE-sulfatase paralogs (MarpuDRAFT_1558). It is unclear whether this highly unusual ORF is a sequencing artifact, a pseudogene, or whether it is actually expressed.

**Table 2:** DGRs with multiple VRs

**GI of RT**	**Number of VRs**	**Organism**	**Sequence identity [%]**
119356297	2	Chlorobium phaeobacteroides DSM 266	52.7
312115534	2	Rhodomicrobium vannielii ATCC 17100	48.0
308273696	2	uncultured Desulfobacterium sp.	45.3
312962032	2	Pseudomonas fluorescens WH6	43.7
83310593	2	Magnetospirillum magneticum AMB-1	59.8
17230989	2	Nostoc sp. PCC 7120	65.7
83309559	2	Magnetospirillum magneticum AMB-1	70.6
186684985	2	Nostoc punctiforme PCC 73102	81.3
126660098	2	Cyanothece sp. CCY0110	47.7
126659397	2	Cyanothece sp. CCY0110	63.4
189425132	2	Geobacter lovleyi SZ	52.1
336435571	2	Lachnospiraceae bacterium 1_4_56FAA	49.3
291612675	2	Sideroxydans lithotrophicus ES-1	62.1
187929429	2	Ralstonia pickettii 12 J	51.8
167841733	2	Burkholderia thailandensis MSMB43	53.6
238027140	2	Burkholderia glumae BGR1^§^	55.6
350551816	2	Thiorhodospira sibirica ATCC 700588	51.8
345871758	2	Thiorhodococcus drewsii AZ1	55.2
355363092	2	Desulfobacter postgatei 2 ac9	72.0
340788559	3	Collimonas fungivorans Ter331^§^	47.5 - 52.3
344343882	3	Marichromatium purpuratum 984	N/A
194337359	4	Pelodictyon phaeoclathratiforme BU-1	48.0 - 57.2
299067086	4	Ralstonia solanacearum CMR15^§^	41.1 - 59.6
347538767	4	Pseudogulbenkiania sp. NH8B	45.2 - 72.1

Organisms marked with ^§^ do not feature additional paralogous ORFs independent of the DGR.

Within each DGR, multiple target ORFs show high protein sequence homology to each other. Also at the nucleotide level, the ORFs fulfill the criteria of paralogy (30% sequence identity over at least 60% of the sequence, [27]) so that gene duplication is the likely mechanism of multiple VR-DGR formation. Notably, even the most distant target ORFs display the hallmarks of continuing and independent diversification (i.e. A exchanges without accumulation of B mutations, and different VR sequences in paralogous ORFs).

Duplication of genes is not an uncommon event in nature. In most cases, there is no significant increase in fitness and one of the copies becomes inactive and is finally deleted again [28]. If duplication proves to be advantageous to the host, both open reading frames are kept as paralogs. The paralogous gene can increase host fitness simply be raising the expression level of the encoded protein, but most often it is associated with neofunctionalization or subfunctionalization [29,30]. This process can be significantly accelerated by combining gene duplication with DGR activity, leading to parallel diversification of a whole protein family and thus a superior means to adapt to environmental demands. However, if all members of a gene family are mutated simultaneously, essential functions might be lost. Consequently, we checked for the presence of additional paralogs in organisms featuring multiple VRs by using one of the respective target proteins as a query for a blastp search. In all but three cases, we found at least one additional paralog without a VR. Thus the diversified genes in multiple VR DGRs are usually part of a bigger gene family and co-exist with more stable counterparts of similar function which act as conserved “ancestor” genes.

Interestingly, our search for paralogous target genes in the complete genomes of the host organisms also unearthed additional ORFs that include perfect variable repeats differing exclusively in A-positions from their corresponding TR. The maximum distance between a DGR RT and additional target ORFs was observed in *Pseudogulbenkiania* sp. NH8B with > 370 kb. Further examination revealed the presence of a strongly mutated RT gene in the vicinity of these distal target ORFs, suggesting that a DGR underwent duplication and lost one of the RTs because the remaining enzyme was sufficient to support diversification of all VRs. Generally, these additional target ORFs were found on different contigs or further than 5 kb from the RT, so that our program could not automatically identify them. However, the program’s ability to identify DGRs per se does not seem affected by this limitation. This is due to the fact that all DGRs that we have found so far contain a “core” DGR cassette comprising 2–4 kb, which is easily covered by the ~ 11 kb input sequence. In order to obtain a quantitative assessment of DGRs with multiple VRs, it would be necessary to run the program on whole genome data. While the length of the analyzed sequence can be increased in DiGReF, this significantly increases the computation time and was therefore not done in this initial study.

### A new structural DGR type features inversions

During our studies, we identified three RTs (*Shewanella baltica* OS155, GI 126090247; *Vibrio* sp. RC586, GI 262403399; *Photobacterium angustum* S14, GI 90580666) that represent a previously unknown structural DGR type. These “inverted” DGRs (Figure 7, Group 4) consist of an RT ORF on one DNA strand, and TR, VR and target ORF on the other DNA strand. Except for the separation of the cassette components on two strands, these elements show all standard features of DGRs such as long repeats (130–139 nt) and a high mutation rate (18–21 A substitutions). Since our program only analyzes the DNA strand coding for the RT, repeats of these “inverted” DGRs cannot be recognized by a standard DiGReF search looking for A-specific mutations. We incidentally found them when we were investigating whether DGRs can only mutate adenine residues. We changed the program to search for repeats with C, G, or T substitutions in the vicinity of RT sequences. For Cs and Gs, we did not find a single hit that matched the search criteria, but for Ts, we found three hits representing the complementary strands of inverted DGRs. Phylogenetically, their RT sequences cluster in one group (Figure 2), suggesting that the inversion was a one-time event that subsequently got distributed to different species via HGT. Though a rare event, the inversion proves that unlike for example retrotransposons, the RT mRNA and the template RNA are not required to form a single transcriptional unit. Theoretically, it might even be possible that TR and VR lie on opposite strands. Indeed, the DGR of *Pseudogulbenkiania* sp. NH8B has four associated VRs, two on the same strand as RT and TR, and two in further distance and on the opposite strand. Thus, the analysis of DGR structures has uncovered two mechanistic aspects of DGR-mediated mutagenesis: transcriptional separation of RT and TR expression, and spatial uncoupling of DGR expression and VR targeting.

## Conclusion

The program DiGReF is designed to easily and automatically search for DGRs. With this program, we were able to reliably identify all previously described DGR sequences, but in addition, we found over 100 new cassette structures that show the typical features of DGRs. Changing the search parameters allowed us to identify new structural DGR types. Currently, the program is mainly limited by incomplete or misassembled sequence data, but allows facile constant surveillance of newly sequenced genomes for DGRs.

Moreover, the modular nature of DiGReF and its flexible output (e.g. in graphical format) greatly facilitate the downstream analysis of various aspects of DGRs. In this work we have analyzed repeat length, nucleotide substitution patterns, RT phylogenetics, cassette structure and interspecies transfer of DGRs, but the program output also offers the possibility to address other questions pertaining to DGR function. For example, the program can be adapted to extract the target ORFs of DGRs. Although the crucial role of DGRs in phage tropism switching is well understood, the function of these elements in bacteria is still unclear. Many target ORFs are located in the membrane, belong to the FGE-sulfatase superfamily and assume a Clec-type fold [31,32], but their exact function is unknown. A systematic large scale comparison of the target proteins may provide insights into which proteins are good targets for DGRs and help to define their biological role. Similarly, DiGReF facilitates the search for accessory proteins and allows detailed analysis of integration determinants such as the IMH region (initiator of mutagenic homing) or the hairpin/cruciform structure downstream of the VR that is required for target site recognition in a subset of DGRs [8]. Thus the software will be a valuable tool for obtaining deeper insights into the function of these unique intriguing retroelements.

## Methods

### RT sequence collection

Using eight protein sequences (GenBank GI-no. 186684985, 134299090, 148359926, 113474819, 42527768, 90580666, 149833092, 41179367) representing RTs from previously described DGRs as queries, we performed psi-blast searches with two iterations against the nr protein database (November 2011). For subsequent iterations, the top thirty hits from the first search were used as queries. More than two iterations did not lead to significant changes in the obtained set of reverse transcriptases. After the last iteration, all hits with an E-value lower than 0.005 were pooled (2651 hits) and used for further analysis.

### Program design

A program (DiGReF) (Additional file 1) was designed to find potential VR and TR sequences. It was written in Perl (ActivePerl [33]) using the BioPerl package [34]. The program retrieves the nucleotide sequences containing RTs from the NCBI GenBank database. A region consisting of the RT-coding sequence and sequences to either side (default length 5 kb to each side) are searched for potential TR/VR pairs. Sliding windows (default size 50 nt, stepsize 1 nt) are considered as candidates for TRs and screened for repeats that match all non-A bases of the whole region containing the RT gene. Hits then are extended to generate the maximum repeat length in which all non-A bases match. In a filtering step, repeats that contain few As in the TR (default: less than 10) and few A-specific substitutions in the VR (default: less than 7) are discarded from the dataset. Alignments of the potential TR/VR pairs are output to a file. A second program module (Additional file 3) converts the results of DiGReF to the GenBank DNA format with the RT and potential TR/VR pairs shown as features. This file can be opened with a sequence viewer program (e.g. Artemis, [11,35]) to allow simple visual assessment of the relative positions of the RT and the TR/VR pair.

### Sequence alignment

Multiple Alignment of the RT sequences from DGRs and other retroelements was performed using MAFFT at the European Bioinformatics Institute [36,37] and COBALT [38]. Sequence Logos were created using WebLogo [39-41].

### Phylogenetic analysis

Phylogenetic analysis was carried out using MEGA5 [42], RAxML [43], and PHYLIP [44]. Trees were constructed using the neighbor joining algorithm with the JTT distance matrix [45] and 1000 bootstrap replications were carried out to give a consensus tree. For comparison, a maximum likelihood tree was also constructed with 1000 bootstrap replications, but gave essentially the same clade pattern. 16S rRNA sequences of the organisms were downloaded from the SILVA database [46,47]). For analysis of distribution of DGRs across prokaryotic classes, the counts for sequenced genomes per class were retrieved from NCBI’s Taxonomy database [48].

## Abbreviations

DGR: Diversity generating retroelement; DiGReF: Diversity generating retroelement finder; RT: Reverse transcriptase; TR: Template repeat; VR: Variable region; ORF: Open reading frame; A, C, T, G: Adenine, cytosine, thymine, guanine; B: C, T, or G.

## Competing interests

The authors declare that they have no competing interests.

## Authors’ contributions

ML developed most of the software components. TS performed most of the data analysis and contributed to writing the manuscript. JYC compiled the RT sequences, participated in software development, and helped with phylogenetic analysis and sequence alignment. NZ and JC conceived of the study and participated in its design. JC contributed to phylogenetic and statistical data analysis and helped to draft the manuscript. NZ carried out RT analysis, coordinated the study and drafted the manuscript. All authors read and approved the final manuscript.

## Supplementary Material

Additional file 1 **Program DiGReF.** Software to search for DGRs in a list of sequences supplied as GI numbers. Requires BioPerl to run. Click here for file

Additional file 2 **Results from BLASTp search and DiGReF analysis.** Part A of the table lists the gi-numbers of all RTs from the psi-blast search that were positive in a DiGReF analysis with default settings (cut off seven or more adenine exchanges in a 50 bp window. Part B shows the additional 47 hits obtained when lowering this cut off to five or more A substitutions. Only six of these are most likely DGRs (i.e. they feature a (L/I/V)GxxxSQ or (L/I/V)GxxxNQ sequence, and their VR is part of an ORF and not a low complexity repeat). Part C lists the remaining gi-numbers of RTs from the psi-blast search that yielded no hit in the DiGReF analysis. Click here for file

Additional file 3 **Program ConvertGB.** Software to convert the output from DiGReF into GenBank format. Requires BioPerl to run. Click here for file

Additional file 4 **Complete NJ tree of DGR RTs.** Protein sequences of DGR RTs were aligned using COBALT and a Neighbor-Joining tree was built with PHYLIP. Bootstrap values >50 are indicated. Phylogenetic groups of organisms that also cluster on RT level are marked. Distances are indicated as expected substitutions per site. A group II intron reverse transcriptase from *Bacillus halodurans* (GI 47076650) was used as outgroup to root the tree. Click here for file

Additional file 5 **NJ tree of 16S rRNAs from organisms featuring DGRs.** 16S RNA sequences were collected from SILVA database if available. A Neighbor-Joining tree was built using MEGA5. Distances are indicated as expected substitutions per site. Click here for file

Additional file 6 **Alignment of DGR RTs.** MAFFT alignment of the 155 DGRs RTs (yellow) identified in this study. For comparison with other known RTs, RTs from 8 group II introns (pink), 8 non-LTR retrotransposons (blue), 9 retroviridae (purple) and 8 telomerases (green) were also included. Conserved domains are indicated as black bars above the alignment. Conserved amino acids are highlighted with colors reflecting their chemical properties. Click here for file

Additional file 7 **DGR RTs contain a positively charged region at their C-terminus.** Additional file a 5 shows a section of Additional file 4 comprising the region C-terminal to domain 5. Only positively charged amino acids are highlighted in red. In DGR RTs, domain 7 is often followed by a patch with high positive charge (up to 11 positively charged amino acids in a 20 amino acid region), a feature that is not found in other RT enzymes. Click here for file
