# Supplementary figures and images for: Analysis of a comprehensive dataset of diversity generating retroelements generated by the program DiGReF
Source: BMC Genomics. 2012 Aug 28;13:430. doi: 10.1186/1471-2164-13-430 (PMC3521204; doi:10.1186/1471-2164-13-430)

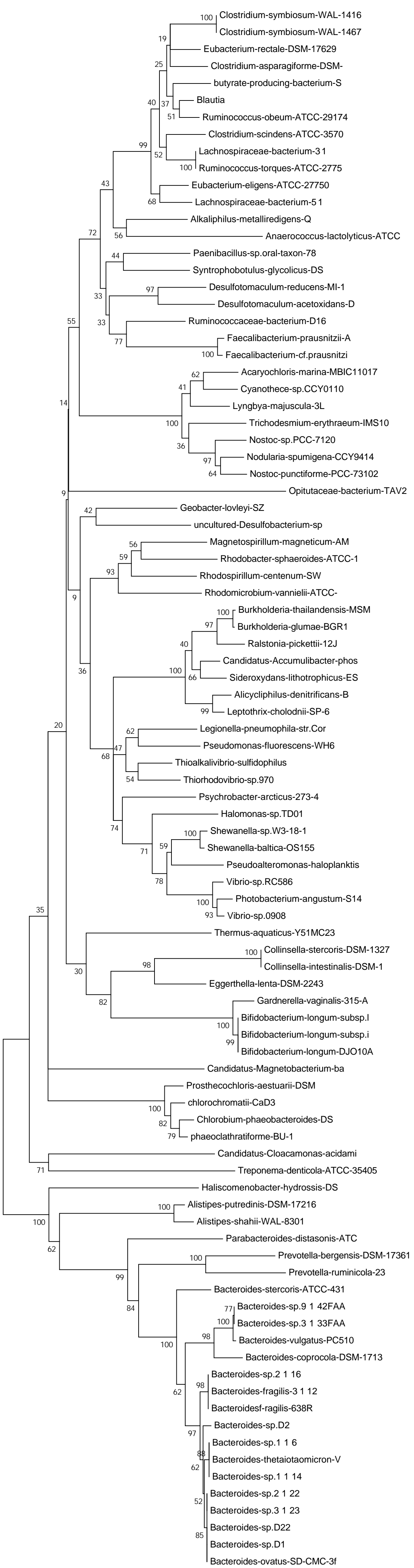

0.02

Supplement: Additional file 5 — NJ tree of 16S rRNAs from organisms featuring DGRs. 16S RNA sequences were collected from SILVA database if available. A Neighbor-Joining tree was built using MEGA5. Distances are indicated as expected substitutions per site. [file 1471-2164-13-430-S5.pdf]

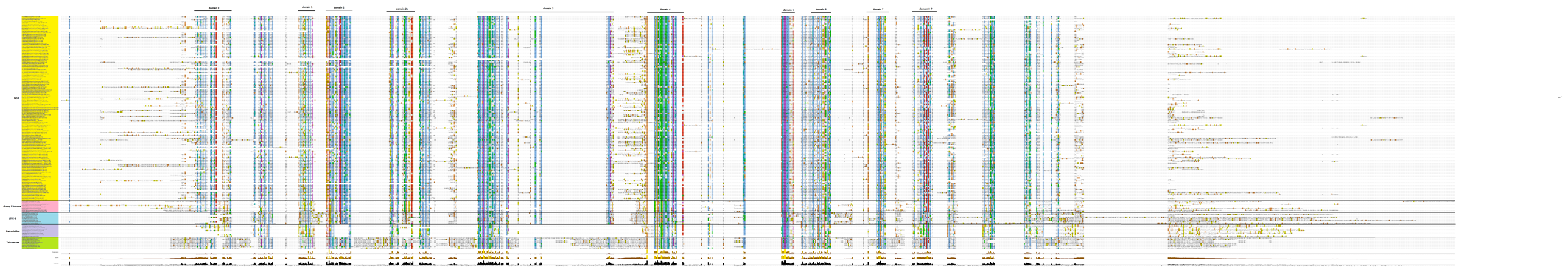

Supplement: Additional file 6 — Alignment of DGR RTs. MAFFT alignment of the 155 DGRs RTs (yellow) identified in this study. For comparison with other known RTs, RTs from 8 group II introns (pink), 8 non-LTR retrotransposons (blue), 9 retroviridae (purple) and 8 telomerases (green) were also included. Conserved domains are indicated as black bars above the alignment. Conserved amino acids are highlighted with colors reflecting their chemical properties. [file 1471-2164-13-430-S6.png]

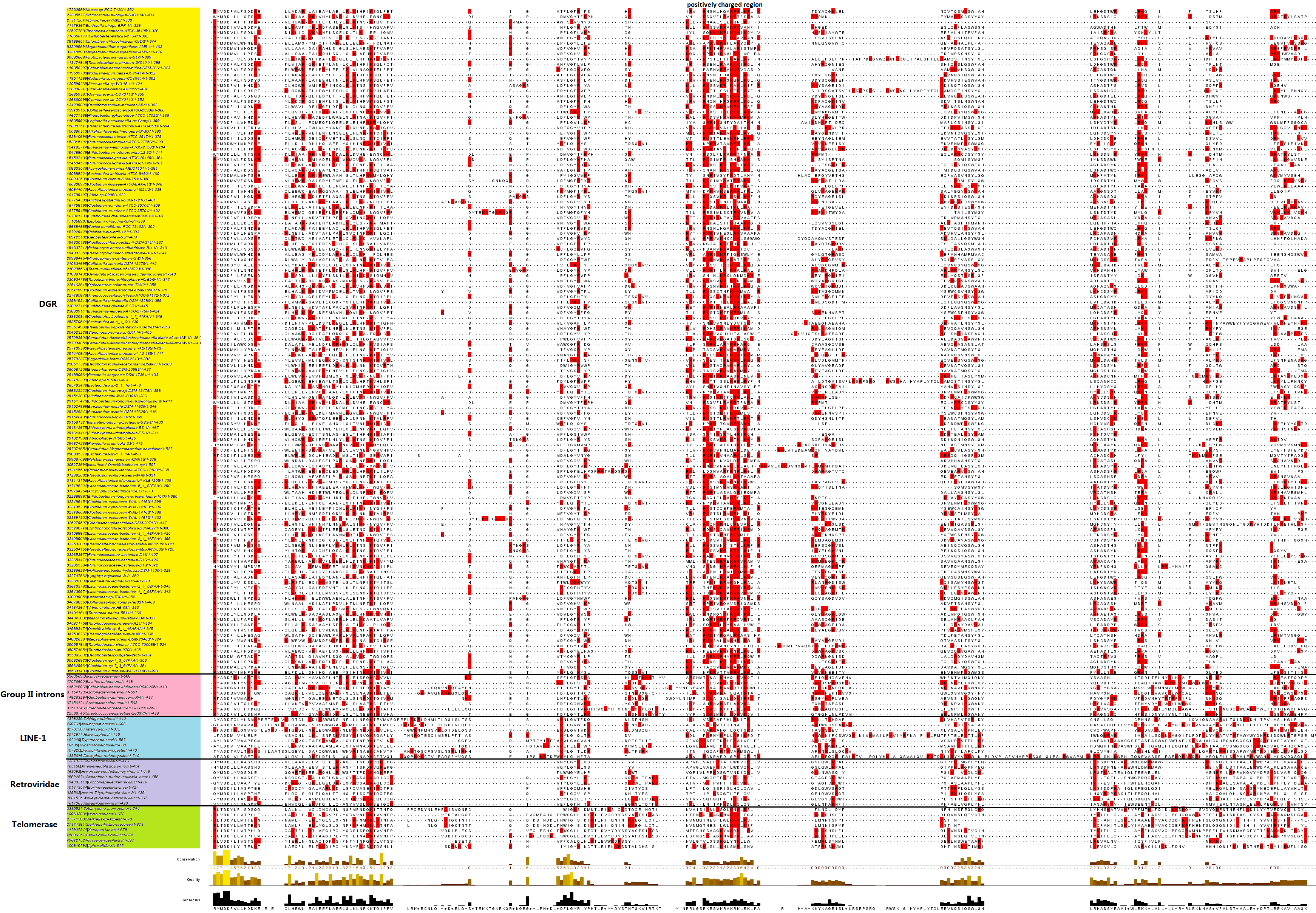

Supplement: Additional file 7 — DGR RTs contain a positively charged region at their C-terminus. Additional file a 5 shows a section of Additional file 4 comprising the region C-terminal to domain 5. Only positively charged amino acids are highlighted in red. In DGR RTs, domain 7 is often followed by a patch with high positive charge (up to 11 positively charged amino acids in a 20 amino acid region), a feature that is not found in other RT enzymes. [file 1471-2164-13-430-S7.png]
